# Evaluation of antifungal and antimycotoxigenic activity of selected natural antioxidants against *Fusarium culmorum* : *in vitro* and *in planta* assays

**DOI:** 10.1002/jsfa.70530

**Published:** 2026-02-16

**Authors:** Safa Oufensou, Davide Fabbri, Maria Antonietta Dettori, Paola Carta, Emanuela Azara, Valeria Ugone, Virgilio Balmas, Ismael Malbrán, Quirico Migheli

**Affiliations:** ^1^ Dipartimento di Agraria Università degli Studi di Sassari Sassari Italy; ^2^ Nucleo di Ricerca sulla Desertificazione Università degli Studi di Sassari Sassari Italy; ^3^ Istituto di Chimica Biomolecolare, Consiglio Nazionale delle Ricerche Sassari Italy; ^4^ Centro de Investigaciones de Fitopatología (CIDEFI‐UNLP‐CIC), Facultad de Ciencias Agrarias y Forestales Universidad Nacional de La Plata Buenos Aires Argentina

**Keywords:** antioxidants, food preservatives, *Fusarium culmorum*, mycotoxins, natural compounds

## Abstract

**BACKGROUND:**

*Fusarium culmorum* causes Fusarium head blight (FHB) in cereals, leading to significant yield losses and contamination with type B trichothecene mycotoxins such as deoxynivalenol (DON) and its acetylated derivatives 3‐ADON and 15‐ADON. Due to the limitations of chemical fungicides, there is increasing interest in sustainable alternatives. Naturally occurring antioxidants have shown potential in modulating fungal growth and mycotoxin production.

**RESULTS:**

This study evaluated the effects of 11 natural antioxidant compounds on *Fusarium culmorum* growth and trichothecene production, both *in vitro* and *in planta. In vitro*: octyl gallate (**7**), honokiol (**9**), and magnolol (**10**) completely inhibited fungal growth and trichothecene production at 0.5 mmol L^−1^. *In planta*: octyl gallate (**7**), reduced the disease index to 0.63% and total mycotoxin levels by 90% while honokiol (**9**), and ascorbic acid (**1**) exhibited notable inhibition in both disease severity and mycotoxin accumulation. The equimolar combination of ascorbic acid and tocopherol demonstrated synergistic inhibition of DON and 3‐ADON *in vitro*, though its efficacy *in planta* was moderate. No correlation was found *in vitro* between the antioxidant or lipophilic properties and antifungal efficacy, whereas *in planta* experiments revealed a positive correlation between antioxidant activity and trichothecene suppression. Statistical analysis was performed using Shapiro–Wilks, Levene and analysis of variance tests (*P* ≤ 0.05).

**CONCLUSIONS:**

Natural antioxidants, particularly octyl gallate and honokiol, represent promising candidates for controlling *F. culmorum* and reducing trichothecene contamination in cereals. Their application could contribute to safer food by reducing trichothecene levels through environmentally friendly strategies. Further research is needed to optimize formulations and assess field‐level efficacy. © 2026 The Author(s). *Journal of the Science of Food and Agriculture* published by John Wiley & Sons Ltd on behalf of Society of Chemical Industry.

## INTRODUCTION

Cereal grains like maize, wheat, and rice are vital staples due to their nutritional value, storability, and efficient cultivation, yet they are vulnerable to physical, chemical, and biological hazards, including mycotoxins.^1^ Among emerging threats, *Fusarium* species, particularly *Fusarium culmorum* (Wm.G. Sm.) Sacc., increasingly affect cereal crops, posing major risks to food safety and production.^2^, ^3^ *Fusarium culmorum*, a soil‐borne fungus, causes diseases such as foot and root rot (FRR) and Fusarium head blight (FHB) of plants, leading to root and stem decay, stunted growth, and decreased grain yield and quality.^4^ The severity of FRR is expected to worsen under drought conditions, especially in regions like Southern Europe, North Africa, and the Middle East, where climate variability is increasing.^5^, ^6^, ^7^

*Fusarium culmorum* produces type B trichothecenes, including deoxynivalenol (DON) and its acetylated derivatives 3‐ADON and 15‐ADON, which are toxic to humans and animals and act as virulence factors in plants.^8^, ^9^

Controlling this pathogen is essential to ensure food safety. Strategies typically involve integrated pest management (e.g., crop rotation, resistant varieties, postharvest storage) and chemical fungicides such as triazoles (metconazole, tebuconazole (1‐(4‐chlorophenyl)‐4,4‐dimethyl‐3‐(1*H*‐1,2,4‐triazol‐1‐ylmethyl)‐pentan‐3‐ol)).^10^, ^11^ However, fungicides pose health and environmental risks and may lead to resistance in fungal populations. Like other fungal pathogens, *Fusaria* can develop resistance to fungicides over time; in fact, repeated use of the same or similar fungicide classes applies selective pressure on the fungal population, allowing resistant strains to survive and become dominant.^12^

In plants, biotic stress stimulates the overproduction of reactive oxygen species (ROS), often called oxidative stress. ROS interact with cellular molecules, leading to protein denaturation, DNA damage, or membrane lipid degradation.^13^

To mitigate the harmful effects of ROS, plants have developed enzymatic and non‐enzymatic antioxidant defence mechanisms.^14^ Antioxidative processes involve enzymes such as ascorbate peroxidase (APX), catalase (CAT), glutathione‐related enzymes like glutathione reductase (GR) and glutathione peroxidase (GPX).^13^ Endogen non‐enzymatic antioxidants are plant secondary metabolites and are classified into lipid‐soluble (e.g., β‐carotene and various tocopherols and tocotrienols, with α‐forms predominant) and water‐soluble reductants (e.g., glutathione, ascorbate, and phenolics).^15^ These endogenous antioxidants scavenge free radicals, preserving cellular integrity and enhancing resistance to fungal invasion.

The application of antioxidant‐based strategies represents a sustainable approach to protect cereal crops and improve food and feed safety, in line with global sustainability and health goals.^16^ However, there are limited studies in the literature evaluating the antimycotoxigenic and antifungal activity of pure antioxidant molecules isolated from natural sources. Notably, natural antioxidants have demonstrated the ability to inhibit a broad spectrum of fungi by disrupting cell membranes, altering fungal metabolism, and interfering with key biochemical pathways.^17^, ^18^

As a result, it is urgent and essential to identify next‐generation inhibitory compounds that can enhance the host plant's defence mechanisms, specifically targeting the pathogenic and mycotoxigenic potential of *Fusarium* spp. rather than its saprophytic phase.

**Figure 1:**
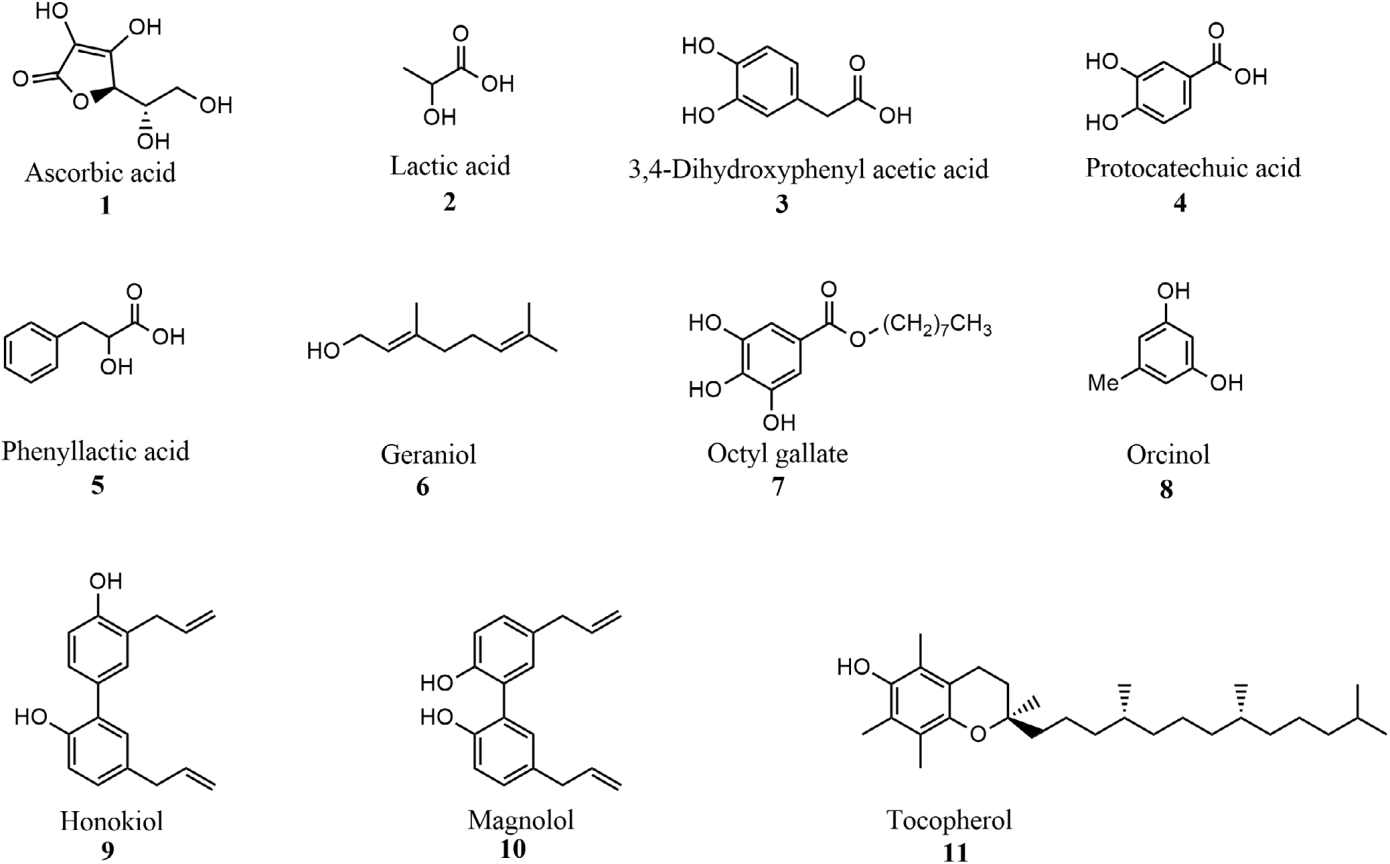
Chemical structure of studied compounds.

This study aimed to evaluate, through both *in vitro* and *in planta* assays, the fungicidal activity and inhibitory effects on trichothecene biosynthesis of 11 natural antioxidants, namely ascorbic acid (**1**), lactic acid (**2**), 3,4‐dihydroxy phenyl acetic acid (**3**), protocatechuic acid (**4**), phenyllactic acid (**5**), geraniol (**6**), octyl gallate (**7**), orcinol (**8**), honokiol (**9**), magnolol (**10**) and α‐tocopherol (**11**) (Fig. 1). The selected compounds were chosen based on their natural origin and GRAS (generally recognized as safe) status, some of them are endogenous compounds presence in durum wheat, they exhibit antioxidant activity and possess different levels of lipophilicity, crucial proprieties in the mechanism of plant defence to prevent pathogen attack. We wanted to study the antioxidant effect of ascorbic acid (**1**) and α‐tocopherol (**11**) against *F. culmorum* and, for the first time, to apply the synergistic effect when an equimolar stoichiometry (1:1 ratio) of these antioxidants have been used against a pathogen. In previous articles we have found that compounds **7**, **9** and **10** ^19^, ^20^ exhibited prior evidence of antifungal and antimycotoxigenic activities and we observed that compound **5** ^21^ showed an encouraging inhibition of fungal growth. Furthermore, we selected compounds for their structural diversity to explore different mechanisms of action against *F. culmorum*.

## MATERIALS AND METHODS

### Fungal strains and culture conditions

The *F. culmorum* wild‐type strain UK99 (Rothamsted Research, Harpenden, UK, NRRL54111) was used for *in vitro* and *in planta* experiments. This strain produces mainly 3‐ADON and lower amounts of DON^22^ and was maintained as described previously.^19^

### Tested compounds

Eleven naturally occurring antioxidant compounds **1**–**11** (Fig. 1) were selected to assess their effect on fungal growth and mycotoxin production *in vitro* and *in planta*. All the compounds and solvents were purchased from Sigma Aldrich (Milan, Italy). All selected compounds are naturally occurring and classified as GRAS ensuring their suitability for agricultural and food‐related applications. Some of the compounds, such as ascorbic acid, α‐tocopherol, and protocatechuic acid, are naturally present in *Triticum durum* and play a role in protecting plant cells from oxidative stress. Their radical scavenging activity was evaluated using the ABTS (2,2′‐azino‐bis‐(3‐ethylbenzothiazoline‐6‐sulfonic acid)) assay, providing a quantitative measure of their antioxidant potential. The compounds' lipophilic properties, estimated via log*P* values, influence their ability to penetrate plant tissues and interact with fungal cells. Several compounds have shown efficacy *in vitro* and *in silico* studies, particularly in inhibiting fungal growth and mycotoxin biosynthesis. The compounds represent a range of chemical classes (vitamins, terpenes, acids, esters, phenols, biphenols), allowing the exploration of different mechanisms of action against *F. culmorum*.

### *In vitro* antifungal activity assay

Eleven naturally occurring compounds (Fig. 1) were tested for their antifungal activity against UK99 in Fusarium minimal medium (FMM).^23^ Each phenolic compound was resuspended in (0.1%) solution water/gellan (Alpha Aesar, Kandel, Germany) and sonicated at room temperature for 1 h at 80 Hz (Elmasonic P 180 H; Elma Schmidbauer GmbH, Singen, Germany). Solid FMM with sodium nitrate (NaNO_3_) as nitrogen source was distributed into Ø90 mm Petri dishes (15 mL/Petri dish) and amended with each compound at a final concentration of 0.5 mmol L^−1^, except for magnolol (**10**) and honokiol (**9**) which were tested at 0.25, 0.125 and 0.05 mmol L^−1^ concentrations, at 45 °C. Then, 10 μL of the conidial suspension of each strain were spotted onto the centre of the Petri dish. Antifungal activity of each compound was measured after 5 days of growth at 25 °C in the dark and expressed as the colony diameter (percentage relative to control). Four replicates were prepared for each isolate/inhibitor combination, and the experiment was repeated once.

### *In vitro* DON analysis assay

#### Sample preparation

Mycotoxins (DON and its acetylated form 3‐ADON) were extracted from the growing mycelia 14 days post‐inoculation. Six mycelial plugs from actively growing mycelium in the Petri dish, prepared and incubated as previously described, were transferred into 2 mL microcentrifuge tubes (Eppendorf, Hamburg, Germany). Then, 1 mL extraction solvent (acetonitrile/water/acetic acid (*v/v/v*), 79:20:1) was added to each tube and shaken in an orbital shaker (Typ LSR‐V; Adolf Kühner AG, Birsfelden, Switzerland) for 2 h at 180 rpm. Subsequently, supernatants were transferred to chromatography vials and diluted 1:10 with extraction solvent.

### In planta assay

Following the *in vitro* findings, compounds **1**, **7**, **9**, and **11**, as well as the equimolar combination of compounds **1** and **11**, were chosen for additional *in planta* assessments of both the pathogenicity and mycotoxin production of *F. culmorum*. The experiments were carried out in the glasshouse of the Centre for Conservation and Valorization of Plant Biodiversity at the University of Sassari, Italy, within a temperature range of 15 to 30 °C. The *T. durum* cv. ‘Saragolla’, susceptible to FHB, was used and plants were daily irrigated. Six wheat spikes were sprayed with the selected compounds at 4 mmol L^−1^. To improve solubilization and stability of the natural antioxidant compounds, *β*‐cyclodextrin CAVAMAX W7 Pharma (β‐CD; Wacker Chemie Italia, Peschiera Borromeo, Italy) and phytic acid (Sigma‐Aldrich, Darmstadt, Germany) were added at 1.5 μg mL^−1^ and 8 μg mL^−1^, respectively, as coadjutants *in planta* experiments.^24^

A treatment with tebuconazole (4 mmol L^−1^) was included. A total of 2 mL of the corresponding compound solution was sprayed on each spike. Treated spikes were spray‐inoculated with 250 mL of 10^4^ CFU mL^−1^ of UK99 inoculum 24 h after the antifungal treatment and subsequently covered with a plastic bag for 48 h to create adequate moisture for infection. Four replicates were performed for each compound and the antifungal treatment was repeated three times, at 1 week interval. Disease index,^25^ grain production (in grams), and total DON (DON + 3‐ADON) production were evaluated. Trichothecenes were quantified by liquid chromatography–mass spectrometry (LC–MS) analysis.

### DON analysis in infected wheat

#### Sample preparation

Wheat seeds (5.0 g) were finely ground and transferred into a 50 mL centrifuge tube with 10 mL of water; 10 mL of 10% (*v/v*) acetic acid in acetonitrile was added and vortexed for 1 min at high speed. Phenomenex extraction kit KSO‐8909 (Agilent Technologies, Santa Clara, CA, USA) (4.0 g magnesium sulphate (MgSO_4_), 1.0 g sodium chloride (NaCl), 1.0 g sodium citrate tribasic dihydrate (SCTD) and 0.5 g sodium citrate dibasic sesquihydrate (SCDS)) was added and hand‐shaken for 1 min. The resulting mixture was centrifuged at 4000 rpm for 5 min, and the supernatant transferred into a 15 mL centrifuge tube containing KSO‐8924 purification kit (900 mg MgSO_4_, 150 mg Primary Secondary Amine [PSA]). The mixture was then shaken over a vortex mixer (3000 rpm for 30 s) and centrifuged at 4000 rpm for 5 min. An aliquot (2 mL) of the supernatant was evaporated to dryness under a nitrogen gas stream and reconstituted in 1 mL of LC–MS mobile phase (A: 5 mmol L^−1^ ammonium acetate with 0.5% acetic acid, B: 5 mmol L^−1^ ammonium acetate in methanol with 0.5% acetic acid). The extracts were filtered through 0.22 m PTFE syringe filters prior to LC–MS analysis.

### Determination of antioxidant activity and estimation of lipophilicity of compounds **1**–**11**

The antioxidant properties of the proposed compounds **1**–**11** were determined by ABTS assays. A 5 mmol L^−1^ stock solution was prepared in absolute ethanol for each compound. The radical scavenging activity of the compounds was determined *in vitro* by ultraviolet‐visible (UV‐visible) spectroscopy as reported in the literature.^26^

Lipophilicity, correlated to many other properties such as solubility and permeability, representing the affinity of a compound for the lipid phase of plant tissues (membranes, waxes, cutin, suberin, etc.) was estimated by ChemBioDraw Ultra 13.0 software using the logarithm of the partition coefficient for *n*‐octanol/water (log*P*).

### Liquid chromatography–high‐resolution mass spectrometry (LC–HRMS) analysis

Separations of mycotoxins were performed using a 1200 series high‐performance liquid chromatography (HPLC) system (Agilent Technologies). A Phenomenex Gemini C18 (150 mm × 2.1 mm, 5 μm, 100 A°) column was used for the chromatography separation. The flow rate was 0.400 mL min^−1^ during a 15 min period with an injection volume of 5 μL. Mobile phases were: 5 mmol L^−1^ ammonium acetate with 0.1% acetic acid (A) and methanol with 0.2% acetic acid (B). A linear gradient elution of solvents was applied with the following programme: 0 min, 80% B; 4 min, 45% B. The column was equilibrated for 6 min before each analysis and the columns compartment was maintained at 37 °C. The chromatography procedure was coupled to a Q Exactive™ Orbitrap high‐resolution mass spectrometry (HRMS) system (Thermo Fisher Scientific, San Jose, CA, USA) equipped with heated electrospray ionization probe (HESI‐II; Thermo Fisher Scientific, Bremen, Germany) operating in both positive and negative ion mode. Parameters of the ion source were as follows: spray voltage, 3.5 kV; sheath gas flow rate 45 (arbitrary units); auxiliary gas, 10 (arbitrary units); sweep gas, 3 (arbitrary units); and capillary temperature at 320 °C. Full MS acquisition was performed with resolution power 70 000 FWHM with mass accuracy of 5 ppm. The MS parameters were: AGC target 3e^6^, maximum injection time (IT) 200 ms, and scan range 100–1000 m/z. The Xcalibur™ 3.1.66 software and Trace Finder 3.0 (Thermo Fisher Scientific, San Jose, CA, USA) were used for LC–MS control and data processing, respectively.

Mass deviations were calculated as parts per million [calculated mass − experimental mass)/calculated mass] and were found to be below 5 ppm.

Peaks were identified on the basis of their retention time relative to external standards (*t*_R_) and HRMS spectra. All quantitative data were acquired in Full MS scan mode; if a target compound was present, its precursor ion scan trigged a PRM (parallel reaction monitoring).

### Statistical analysis

The mean and standard error (SE) of at least four replicated measurements were calculated for each parameter. Statistical model's assumptions were checked graphically, using Q‐Q and residuals *versus* fitted values plots, and by means of the Shapiro–Wilks and Levene's tests. FHB disease index, colony diameter relative to the control, and trichothecene production, both *in vitro* and *in planta*, showed a non‐normal distribution of the error and were transformed using the arcsine square root and the log‐10 functions, respectively. One‐way analysis of variance (ANOVA) was performed, and means were compared by Fisher's least significant difference (LSD) test (*P* ≤ 0.05) using Minitab (Minitab Ltd, Coventry, UK) for Windows, release 17.

## RESULTS

Eleven naturally occurring antioxidants from different chemical classes including vitamins (compounds **1** and **11**) terpenes (compound **6**), acids and esters (compounds **2**, **3**, **4**, **5** and **7**), phenols and biphenols (compounds **8**, **9** and **10**) (Table 1) were selected for evaluating their effects on *F. culmorum* vegetative growth, pathogenicity, and mycotoxin production. Lipophilicity (log*P*) of all compounds was estimated by theoretical calculations, and their radical scavenging activity was determined *in vitro* through the ABTS assay and expressed as Trolox equivalent antioxidant capacity (TEAC) (Table 1).

**Table 1:** Lipophilicity values and antioxidant activity of tested compounds

Compound	Log*P* ^a^	ABTS (TEAC)^b^, absolute ethanol, time = 6 min
*Vitamins*		
Ascorbic acid (**1**)	−3.36	1.09 ± 0.05
α‐Tocopherol (**11**)	9.98	1.04 ± 0.02
Ascorbic acid/α‐tocopherol 1:1		0.99 ± 0.01
*Terpenes*		
Geraniol (**6**)	2.49	No activity
*Acids and esters*		
Lactic acid (**2**)	−0.67	No activity
3,4‐Dihydroxyphenyl acetic acid (**3**)	0.76	2.54 ± 0.03
Protocatechuic acid (**4**)	0.81	1.12 ± 0.04
Phenyllactic acid (**5**)	1.16	No activity
Octyl gallate (**7**)	3.6	3.24 ± 0.13
*Phenols and biphenols*		
Orcinol (**8**)	1.74	2.04 ± 0.03
Honokiol (**9**)	5.03	1.11 ± 0.01
Magnolol (**10**)	5.03	0.55 ± 0.06

^a^ Log*P* = lipophilicity estimated by theoretical calculations, which express the partitioning of the phenols in a *n*‐octanol/water system.

^b^ TEAC, Trolox equivalent antioxidant capacity; ABTS, 2,2′‐azino‐bis‐(3‐ethylbenzothiazoline‐6‐sulfonic acid).

Tebuconazole, a triazole fungicide, was used as standard compound. In this study, we chose the lowest concentration previously used in the *in vitro* assay^27^ to identify compounds that could minimize both the pathogenicity and mycotoxin production of *F. culmorum*. Honokiol (**9**) and magnolol (**10**), tested at 0.25, 0.125 and 0.05 mmol L^−1^ and octyl gallate (**7**) tested at 0.5 mmol L^−1^ concentrations proved to be the most effective fungicides, completely inhibiting fungal growth (*F* = 220.67; *P*‐value < 0.0001) (Table 2). In terms of mycotoxin accumulation after 7 days, treatments with octyl gallate (**7**), magnolol (**9**) and honokiol (**10**), reduced both DON (*F* = 785.19; *P*‐value < 0.0001) and 3‐ADON (*F* = 176.42; *P*‐value < 0.0001) production below detectable levels (Table 2).

**Table 2:** Effect of tested compounds **1**–**11** *in vitro* on fungal growth and trichothecene (deoxynivalenol (DON) and its acetylated derivative 3‐ADON) production by *Fusarium culmorum* UK99: treatment mycelial colony diameter control 100 ± 0^a^

Treatment	Concentration (mmol L^−1^)	Fungal growth inhibition (%)	DON (ng g^−1^)	3‐ADON (ng g^−1^)
Control	0.5	0 ± 1.82^a^	0.470 ± 0.0527^a^	1.258 ± 0.1333^a^
Ascorbic acid (**1**)	0.5	3.84 ± 0,54^b^	ND	0.517 ± 0.017^b^
α‐Tocopherol (**11**)	0.5	9.74 ± 5.60^bc^	0.458 ± 0.026^a^	1.095 ± 0.736^ab^
Ascorbic acid (**1**) + α‐tocopherol (**11**)	0.5	16.69 ± 10.93^cd^	ND	ND
Lactic acid (**2**)	0.5	16.79 ± 8.46^d^	ND	ND
3,4‐Dihydroxyphenyl acetic acid **(3)**	0.5	39.09 ± 4.60^f^	ND	0.413 ± 0.498^c^
Protocatechuic acid (**4**)	0.5	6.24 ± 1.97^b^	ND	ND
Phenyllactic acid (**5**)	0.5	28.54 ± 12.85^e^	0.020 ± 0.009^b^	0.077 ± 0.020^de^
Geraniol (**6**)	0.5	32.13 ± 10.58^ef^	0.017 ± 0.001^b^	0.103 ± 0.009^d^
Octyl gallate (**7**)	0.5	100^g^	ND	ND
Orcinol (**8**)	0.5	13.67 ± 3.70^cd^	ND	0.044 ± 0.09^e^
Honokiol (**9**)	0.25	100^g^	ND	ND
Honokiol (**9**)	0.125	100^g^	ND	ND
Honokiol (**9**)	0.05	100^g^	ND	ND
Magnolol (**10**)	0.25	100^g^	ND	ND
Magnolol (**10**)	0.125	100^g^	ND	ND
Magnolol (**10**)	0.05	99.04 ± 2.14^g^	ND	ND

*Note*: ND, not detected. Treatments sharing the same letter are not statistically different (least significant difference (LSD) test).

Lactic acid (**2**), protocatechuic acid (**4**), phenyllactic acid (**5**), geraniol (**6**) and orcinol (**8**) exerted low to moderate impact on fungal growth, with respective inhibition percentages of approximately 17%, 7%, 28%, 32% and 13%, while significantly inhibited the production of both DON and 3‐ADON. 3,4‐Dihydroxyphenyl acetic acid (**3**) suppressed completely DON production while partially affecting 3‐ADON accumulation (33% inhibition) and moderately inhibiting fungal growth (39%) (Table 2).

Ascorbic acid (**1**) and α‐tocopherol (**11**) exhibited minimal antifungal activity, resulting in reductions of fungal growth by approximately 4% and 9%, respectively (Table 2). Their equimolar mixture showed a slight enhancement in fungal growth inhibition compared to the individual compounds (16%) (Table 2).

Ascorbic acid (**1**) completely suppressed DON production and inhibited 3‐ADON (59%). In contrast, α‐tocopherol (**11**) had no effect on DON production and 3‐ADON accumulation was reduced by only 13% compared to the untreated control (Table 3). Notably, the equimolar combination of ascorbic acid (**1**) and α‐tocopherol (**11**) resulted in a profound inhibition of both mycotoxins, reducing DON and 3‐ADON levels below the detection limit (Table 2).

**Table 3:** Effect of selected antioxidants *in planta* alone or in combination, on disease index, grain yield and trichothecene (deoxynivalenol (DON) and its acetylated derivatives 3‐ADON and 15‐ADON) production by *Fusarium culmorum* UK99 in durum wheat (cv. Saragolla)

Treatment	Disease index (%)	Seeds yield (g)	DON (ng g^−1^)	3‐ADON (ng g^−1^)	15‐ADON (ng g^−1^)	Total mycotoxins (ng g^−1^)
Control	13.63 ± 7.80^c^	6.56 ± 1.07	3875.74 ± 3.432.58^a^	263.51 ± 271.55^a^	545.82 ± 537.53^a^	4685.17 ± 4.240.90^a^
Tebuconazole	1.65 ± 1.91^a^	9.59 ± 2.42	32.11 ± 20.83^d^	2.22 ± 2.04^c^	5.84 ± 3.65^c^	40.16 ± 22.04^d^
Ascorbic acid (**1**)	4.18 ± 5.01^a^	9.70 ± 3.75	2.227.65 ± 2.578.24^abc^	113.03 ± 146.98^ab^	233.68 ± 292.29^ab^	2574.35 ± 3.013.08^abc^
α‐Tocopherol (**11**)	4.15 ± 1.70^ab^	8.36 ± 2.38	1.947.42 ± 1.795.11^abc^	131.92 ± 131.90^ab^	281.22 ± 275.17^ab^	2360.56 ± 2.197.26^abc^
Tocopherol (**11**) + ascorbic acid (**1**)	10.00 ± 2.96^bc^	7.07 ± 2.07	1.724.26 ± 734.88^ab^	72.85 ± 30.85^ab^	158.29 ± 71.59^ab^	1955.40 ± 802.54^ab^
Octyl gallate (**7**)	0.83 ± 1.65^a^	9.08 ± 2.26	391.84 ± 334.41^c^	16.24 ± 8.93^b^	37.93 ± 19.59^b^	446.02 ± 350.33^c^
Honokiol (**9**)	3.33 ± 2.74^a^	8.98 ± 2.49	855.56 ± 884.46^bc^	47.64 ± 61.59^b^	8.32 ± 122.49^b^	1007.29 ± 1.061.17^bc^

*Note*: Values are expressed as mean values (± standard error). Treatments sharing the same letter are not statistically different (least significant difference (LSD) test). Tebuconazole has not been included in the statistical analysis to better observe the difference between the treatments.

**Figure 2:**
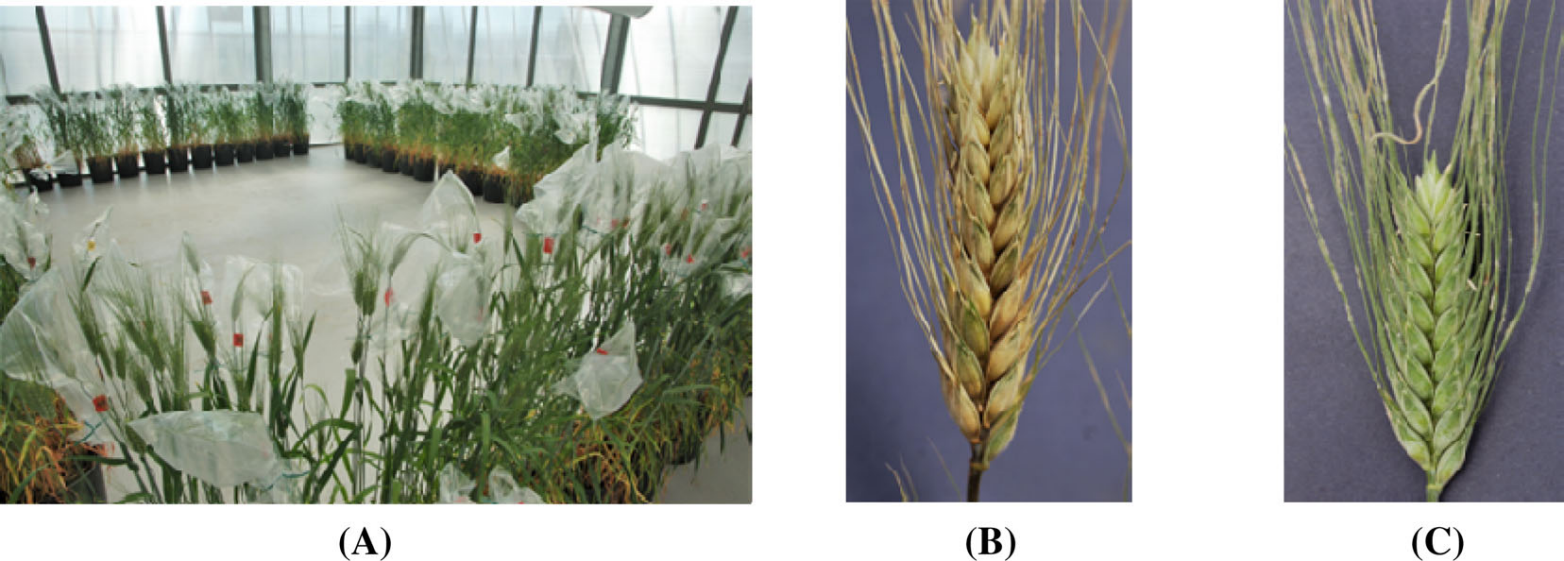
(A) *In planta* experiments at Centre for Conservation and Valorization of Plant Biodiversity; (B) control; (C) treatment with octyl gallate (**7**), at 4 mmol L^−1^ concentration.

Based on *in vitro* results, compounds **1**, **7**, **9**, and **11**, along with the equimolar mixture of compounds **1** and **11** were selected for further evaluation of their effects on mycotoxin biosynthesis *in planta* (Table 3 and Fig. 2(A)). Except for the combination of ascorbic acid (**1**) and α‐tocopherol (**11**), all treatments significantly reduced FHB, when compared to the control (13.63%) (*F* = 6.46; *P*‐value = 0.0002) (Table 3 and Fig. 2(B),(C)). Treatment with octyl gallate (**7**) at a concentration of 4 mmol L^−1^, resulted in the lowest disease index (0.83%).

Honokiol (**9**) also demonstrated significant efficacy, reducing the disease index to about 3%.

(Table 3). Ascorbic acid (**1**) and α‐tocopherol (**11**), when applied individually, exhibited comparable protective effects (approximately 4%), leading to a disease index reduction of 30% relative to the control (Table 3). Conversely, the equimolar combination of ascorbic acid (**1**) and α‐tocopherol (**11**) displayed a disease index of 10%, which was only slightly lower than the control value (Table 3).

Wheat spike analysis revealed that all tested compounds enhanced seed production compared to the control, though the differences were not statistically significant (*F* = 0.99; *P*‐value = 0.4558). Markedly, spikes treated with 4 mmol L^−1^ ascorbic acid (**1**) produced more seeds (9.70 g) than the control (6.56 g) and those treated with tebuconazole (9.59 g) (Table 3).

Only honokiol (**9**) and octyl gallate (**7**) significantly reduced DON (*F* = 6.66; *P*‐value = 0.0005), 3‐ADON (*F* = 5.49; *P*‐value = 0.0015), 15‐ADON (*F* = 5.72; *P*‐value = 0.0012), and total trichothecene contaminations (*F* = 6.63; *P*‐value = 0.0005) when compared with the control (Table 3). Octyl gallate (**7**) reduced total trichothecene contamination (DON + 3‐ADON + 15‐ADON) by approximately 90% compared to the untreated control and its inhibitory activity was only ten times lower than that observed for tebuconazole (Table 3). Ascorbic acid (**1**), α‐tocopherol (**11**) and honokiol (**9**) reduced total mycotoxins by 45%, 50% and 79%, respectively. The equimolar mixture of ascorbic acid (**1**) and α‐tocopherol (**11**) exhibited a higher reduction (58%) compared to the two compounds alone (Table 3).

## DISCUSSION

This study evaluated the antifungal and antimycotoxigenic of 11 selected naturally occurring antioxidants (compounds **1**–**11**) against *F*. *culmorum* both *in vitro* and *in planta*. Compounds exhibited varying degrees of inhibition on fungal growth and trichothecenes production. Although lactic acid (**2**) and phenyllactic acid (**5**) are not classical antioxidants, they exert indirect antioxidant effects by modulating cellular metabolism and redox homeostasis in *Fusarium graminearum*.^28^ Geraniol (**6**), an oxygenated monoterpene naturally present in various spice and food plants, is recognized for its potent antioxidant activity, including suppression of lipid peroxidation, ROS, and nitric oxide (NO) formation.^29^ However, it exhibits no scavenging ability against the ABTS ion in an alcoholic solvent. Most of the selected compounds **1**–**11** are plant‐derived metabolites commonly found in food, spices, or food preservatives^30^, ^31^ and all are classified as GRAS. Remarkably, ascorbic acid (**1**), α‐tocopherol (**11**) and protocatechuic acid (**4**) are also endogenous antioxidants present in durum wheat (*T. durum*)^13^, ^15^ that play key roles in protecting plant cells from oxidative damage. Ascorbic acid (**1**) helps detoxify ROS^32^, ^33^ while α‐tocopherol (**11**) maintains cell membrane integrity under stress conditions.^34^ Although protocatechuic acid (**4**) is known for its antioxidant properties in various plants, studies confirming its presence as an endogenous antioxidant in durum wheat are limited.^35^ An effective approach in this context is the exploration of antioxidant combinations that enhance the efficacy of conventional antioxidants.^36^ For example, an equimolar blend of α‐tocopherol (**11**) and ascorbic acid (**1**) demonstrated a synergistic interaction, significantly amplifying their antioxidant potential.^37^ In our *in vitro* assay's ascorbic acid (**1**) and α‐tocopherol (**11**) exhibited limited antifungal activity but ascorbic acid (**1**) showed significant effects on mycotoxin suppression. Despite their markedly different chemical structures, compounds **1** and **11** exhibit similar radical scavenging capacities based on TEAC values (Table 1) though their differing lipophilicity may influence their efficacy in inhibiting DON production. Ascorbic acid (**1**) exerts its antioxidant effects primarily by donating a hydrogen atom to lipid radicals, stabilizing them and thereby preventing oxidative damage. Importantly, ascorbic acid (**1**) is essential for regenerating α‐tocopherol (**11**) from its tocopheroxyl radical, thus sustaining the antioxidative function of vitamin E.^37^, ^38^ Given these complementary roles, it was of considerable interest to determine whether α‐tocopherol (**11**) could synergistically interact with ascorbic acid (**1**) to mitigate mycotoxin accumulation. *In vitro* assay demonstrated that a 1:1 equimolar mixture of α‐tocopherol (**11**) and ascorbic acid (**1**) achieved complete inhibition (100%) of both DON and 3‐ADON production at a total concentration of 0.5 mmol L^−1^, while exerting only a modest (16%) inhibition on fungal growth. These results indicate a clear synergistic interaction between the two antioxidants that selectively target mycotoxin biosynthesis without significantly compromising fungal viability. This selective inhibition suggests that the combination modulates specific pathways related to mycotoxin production possibly through stabilization of cellular redox balance without broadly affecting the general metabolic processes required for fungal growth.

The antifungal and antioxidant properties of gallic acid esters have been previously linked to their alkyl chain length, which enhances membrane permeability and bioactivity.^39^ Among the tested acids and esters, octyl gallate (**7**) demonstrated *in vitro* the highest activity, exhibiting superior log*P* and TEAC values, completely inhibiting the fungal growth and trichothecene production. A significant reduction in trichothecene biosynthesis was observed also when the substrate was supplemented with lactic acid (**2**), protocatechuic acid (**4**), and phenylacetic acid (**5**) at 0.5 mmol L^−1^ concentration, although their fungicidal activity remained modest. Notably, 3,4‐dihydroxyphenylacetic acid (**3**) led to almost complete inhibition of DON and partial inhibition of 3‐ADON while also reducing fungal growth. When *F*. *culmorum* was cultured in the presence of geraniol (**6**), vegetative growth was notably reduced compared to the untreated control, and the production of DON and 3‐ADON was strongly inhibited. Among the tested *in vitro* compounds, orcinol (**8**) slightly reduces the fungal growth while completely inhibits DON and 3‐ADON trichothecene production. Orcinol (**8**), a natural phenolic compound derived from lichens, with known antimicrobial and radical scavenging abilities.^40^ With its two hydroxyl groups on the aromatic ring, compound **8** exhibits significantly lower lipophilicity and a slightly higher radical scavenging capacity compared to honokiol (**9**) and magnolol (**10**). Building on our previous study,^27^ we evaluated the antifungal efficacy of both magnolol (**10**) and its structural isomer honokiol (**9**) *in vitro* at three concentrations: 0.25, 0.125, and 0.05 mmol L^−1^ showing near‐complete inhibition of fungal growth at all levels highlighting their strong antifungal potential. Despite being structural isomers, honokiol exhibited twice the radical scavenging activity of magnolol, likely due to the absence of intramolecular hydrogen bonding that limits proton donation in magnolol.^41^ Molecular docking analyses on the three‐dimensional (3D) model of *F*. *culmorum* TRI5^27^ revealed that honokiol (**9**), due to its greater structural flexibility, interacts predominantly with amino acid clusters responsible for inhibiting trichothecene production. Both compounds share similar lipophilicity, supporting their comparable *in vitro* efficacy.

Based on the *in vitro* results, we selected compounds **1**, **7**, **9**, and **11** and an equimolar mixture of compounds **1** and **11** for further investigation *in planta*. Wheat spike analysis demonstrated that all tested chemical treatments improved seed yield relative to the untreated control. Among the treatments, the application of ascorbic acid (**1**) led to the highest seed production achieved by a single component, surpassing the performance of tebuconazole‐treated spikes suggesting its dual role in oxidative stress mitigation and plant growth promotion. The findings highlight the potential of ascorbic acid (**1**) as an alternative or complementary treatment to conventional fungicides like tebuconazole in optimizing wheat productivity. Treatment with α‐tocopherol (**11**) improved seeds yield and reduced disease index, though to a lesser extent. Both compounds **1** and **11** significantly reduced the disease index. The application of an equimolar mixture of ascorbic acid (**1**) and α‐tocopherol (**11**) resulted in lower grain yield and disease index compared to individual treatments indicating potential antagonistic effects *in planta*. However, this combination reduced total mycotoxin levels by 58%, suggesting a mild synergistic interaction between the two antioxidants. Ascorbic acid's role in regenerating α‐tocopherol (**11**) from its oxidized form may contribute to this interaction. The differing efficacy of compounds *in vitro* and *in planta* in reducing mycotoxin production highlights the complexity of antifungal activity in different environments. *In vitro* conditions provide a direct interaction between the fungal pathogen and the inhibitor, with both being uniformly distributed in the same medium. However, *in planta*, the dynamics change significantly, as the compounds must penetrate plant tissues to reach the pathogen. Hence, their efficacy is influenced by several factors, including lipophilicity, antioxidant activity, and the composition of the carrier solution. Lipophilicity determines how well the compound can move across plant cuticle and cell wall while antioxidant activity may influence plant defence mechanisms. These combined factors explain the observed differences in efficacy between *in vitro* and *in planta* conditions and highlight the complexity of translating laboratory findings into practical field applications. Among the tested compounds, octyl gallate (**7**) demonstrated the highest efficacy, resulting in increased seed yield and significantly reducing both the disease index and total trichothecene contamination compared to the untreated control. Honokiol (**9**) also showed a strong ability to reduce total mycotoxin levels and demonstrated notable effectiveness in increasing grain yield and lowering the disease index. The formulation of the carrier solution also plays a crucial role in enhancing compound solubility, stability, and transport within plant tissues. These findings suggest that while octyl gallate (**7**) and honokiol (**9**) exhibit strong antifungal and mycotoxigenic activities *in vitro*, their practical application *in planta* requires further optimization. *In vitro* assays provide a controlled and homogeneous medium where the interaction between the fungal pathogen and antioxidant compounds occurs directly and uniformly. In contrast, *in planta* experiments are influenced by biological and environmental factors. The compounds must penetrate plant tissues to reach the pathogen, and their efficacy is modulated by plant metabolism, tissue architecture, and external conditions such as temperature and humidity. Furthermore, lipophilicity plays a key role in determining the ability of compounds to traverse plant cuticles and cell walls, thereby affecting their bioavailability and antifungal performance. The composition of the carrier solution also contributes to solubility, stability, and transport of the compounds within plant tissues. These combined factors explain the observed differences in efficacy between *in vitro* and *in planta* conditions and highlight the complexity of translating laboratory findings into practical field applications. By acknowledging these limitations, we aim to provide a realistic perspective on the potential of natural antioxidants and to guide future research toward formulation strategies and delivery systems that can improve their performance under real agricultural conditions.

## CONCLUSION

This study evaluated the effects of 11 natural antioxidants on fungal growth and mycotoxin production both *in vitro* and *in planta*. Octyl gallate (**7**), honokiol (**9**), and magnolol (**10**) were the most effective *in vitro*, while *in planta*, trichothecene production was significantly inhibited by octyl gallate (**7**), honokiol (**9**), α‐tocopherol (**11**), ascorbic acid (**1**), and combination of compounds **1** and **11**. The results suggest that using natural antioxidants alone or in mixtures represents a promising strategy to control *F. culmorum* and to reduce mycotoxin contamination in cereals. The demonstrated efficacy of natural antioxidants in reducing both fungal growth and trichothecene contamination supports their potential use as sustainable alternatives to conventional fungicides. These compounds, being naturally derived and classified as GRAS, offer a safer and environmentally friendly approach to crop protection.

Furthermore, we suggest future research directions focused on optimizing the formulation of these antioxidants for field application. This includes investigating synergistic combinations, improving compound stability and bioavailability through carrier systems, and evaluating their performance under diverse environmental conditions. Such studies will be essential to translate laboratory results into effective and scalable solutions for integrated pest and mycotoxin management in cereal production.

## FUNDING INFORMATION

Projects: ‘NutrAge, Nutrizione Alimentazione ed invecchiamento attivo’ FOE 2021‐D.M. MUR Prot. no. 844 dated 16 July 2021, DBA.AD005.225, CUP B83C21001810005; ‘Fusarium crown rot on durum wheat in southern Italy: epidemiology, evolution and toxigenic potential in a climate change scenario’ – Progetti di Ricerca di Rilevante Interesse Nazionale (PRIN) – Decreto Direttoriale no. 104 dated 2 February 2022, MUR: 2022L5Y28K – ERC Sector: LS9‐CUP: J53D23010140006.

## CONFLICT OF INTEREST

The authors declare there are no conflicts of interest.

## ETHICS STATEMENT

Not applicable.

## AUTHOR CONTRIBUTIONS

Conceptualization: M.A. Dettori and S. Oufensou; methodology: S. Oufensou, V. Balmas, M.A. Dettori and D. Fabbri; validation: S. Oufensou, I. Malbrán, P. Carta and D. Fabbri; formal analysis: E. Azara, S. Oufensou, I. Malbrán, P. Carta, V. Balmas, P. Carta, V. Ugone and D. Fabbri; data curation: E. Azara, S. Oufensou, V. Balmas and P. Carta; writing – original draft preparation: M.A. Dettori and D. Fabbri; writing – review and editing: M.A. Dettori, D. Fabbri, S. Oufensou and Q. Migheli; funding acquisition: M.A. Dettori and Q. Migheli. All authors have read and agreed to the published version of the manuscript.

## Data Availability

The data that support the findings of this study are available on request from the corresponding author. The data are not publicly available due to privacy or ethical restrictions.
